# Three Dimensionally Printed Octacalcium Phosphate via Binder Jetting for Use in Bone Grafting Applications

**DOI:** 10.3390/ijms26125633

**Published:** 2025-06-12

**Authors:** Autcharaporn Srion, Faungchat Thammarakcharoen, Watchara Chokevivat, Waraporn Suvannapruk, Jintamai Suwanprateeb

**Affiliations:** 1 Biofunctional Materials and Devices Research Group, National Metal and Materials Technology Center, National Science and Technology Development Agency, Pathum Thani 12120, Thailand; autchars@mtec.or.th (A.S.); faungcht@mtec.or.th (F.T.); watcharc@mtec.or.th (W.C.); warapors@mtec.or.th (W.S.); 2 Thammasat University Center of Excellence in Computational Mechanics and Medical Engineering, Thammasat University, Pathum Thani 12120, Thailand

**Keywords:** octacalcium phosphate, bone graft, three-dimensional printing, binder jetting

## Abstract

This study investigates the fabrication and bioactivity of monophasic octacalcium phosphate (OCP) constructs using 3D-printed calcium sulfate precursors. A single-step and a two-step process were employed, transforming calcium sulfate into OCP through a controlled phase transformation in a disodium hydrogen phosphate solution. The results revealed that a single-step process for OCP conversion in 3D printed samples was unsuccessful due to incomplete transformation and the formation of intermediate phases such as brushite and monetite. In contrast, the two-step process enabled the efficient production of monophasic OCP in a shorter timeframe. The converted OCP samples exhibited a compressive strength of 7.65 ± 0.46 MPa and a contact angle of zero, indicating adequate handling strength and high wettability. The resorbability of 3D-printed OCP in simulated body fluid (SBF) was evaluated, showing weight loss through gradual dissolution accompanied by the release of calcium and phosphorus ions, followed by the consumption of these ions for reprecipitation back into OCP without direct transformation into hydroxyapatite (HA). Biocompatibility and bioactivity testing demonstrated high cell viability (96.67 ± 0.18%) using the MTT assay, indicating that the 3D-printed OCP was not cytotoxic. Alamar blue and alkaline phosphatase (ALP) activity assay showed that 3D-printed OCP supported preosteoblast proliferation and osteogenic differentiation.

## 1. Introduction

OCP is one of the calcium phosphate families with the chemical formula Ca_8_(HPO_4_)_2_(PO_4_)_4_·5H_2_O. It is recognized as a precursor in the formation of HA (Ca_10_(PO_4_)_6_(OH)_2_), the primary mineral component of bone and teeth, due to its structural and compositional similarity to HA and its ability to transform into HA under physiological conditions [1,2,3,4]. This transformation is of significant interest in biomineralization studies and biomedical applications, particularly in bone regeneration and repair [5]. The unique properties of OCP, such as its layered structure and higher solubility compared to HA, make it an ideal candidate for medical applications. The layered structure enables the incorporation of biological molecules, thereby enhancing their bioactivity and resorbability [6]. Moreover, OCP’s solubility suggests it can serve as a temporary scaffold that gradually converts to more stable HA in physiological environments, supporting new bone ingrowth [7]. OCP promotes osteoconductivity, facilitating the growth and attachment of osteoblasts, and demonstrates osteoinductivity, encouraging the differentiation of progenitor cells into osteogenic cells [8]. The conversion of OCP to HA under physiological conditions not only enhances the scaffold’s stability but also provides a sustained release of calcium and phosphate ions, essential for bone mineralization and growth. This gradual transformation supports the natural remodeling process of bone, ensuring better integration and regeneration.

OCP exists as a metastable form in water and slowly converts into a more stable phase, with the rate and nature of this change varying according to the solution’s pH. Therefore, OCP can be synthesized by wet chemical processes but not by dry processes due to the crystalline water in its composition. Traditionally, OCP is synthesized via precipitation, which involves mixing calcium and phosphate solutions under controlled pH and elevated temperatures to favor OCP formation over other calcium phosphate phases or hydrolysis methods by converting from other calcium phosphates, typically under milder conditions [9,10,11,12,13,14]. Typically, studies conducted on OCP involved synthesizing OCP powders or fabricating composites using OCP powder as a filler, not as solid-shaped OCP structures suitable for implantation. This was related to the water molecules in its structure, causing the decomposition during consolidation by traditional methods involving high-temperature sintering [15]. Recent advancements in 3D printing technology have opened new ways for the application of bone grafting and tissue engineering. Integrating 3D printing technology offers numerous advantages in the fabrication of bone grafts and scaffolds. This method enables the production of bioceramic grafts and scaffolds with tailored geometries and customizable properties [16]. Some 3D printing techniques have already been utilized to fabricate 3D-shaped OCP structures, including digital light processing (DLP), direct ink writing (DIW), and binder jetting; however, research in this area remains limited [17,18,19,20,21,22]. In the case of binder jetting, a major limitation is the relatively poor mechanical strength of the as-printed parts, which is primarily attributed to high porosity and weak interparticle bonding inherent to the binder jetting process [23]. To overcome these limitations, recent research has focused on optimizing powder characteristics to improve packing density and bonding strength by employing a bimodal particle size distribution in binder jetting powders, which significantly increases the density of the printed parts, leading to enhanced mechanical properties, including notable improvements in strength and durability [24].

However, a key advantage of binder jetting is that it enables the fabrication of OCP structures through the layer-by-layer deposition of a liquid binder onto a powder bed at low temperatures without the need for polymer carriers. This contrasts with other 3D printing techniques, such as DLP and DIW, which typically require a polymer matrix as a carrier phase, with OCP used only as a filler [17,18,19]. Binder jetting was found to be a viable method for 3D printing of OCP scaffold [20,21]. These OCP scaffolds could be loaded with plasmid DNA encoding the VEGFA gene, which promotes vascularization, thus facilitating better integration with the host tissue [22]. However, the binder jetting process took at least 14 days to produce the OCP structure, and a phase-pure OCP was still not obtained, containing amounts of residual unreacted brushite, as well as newly formed HA.

Previous studies have demonstrated that binder jetting with calcium sulfate-based powders and water-based binders, combined with low-temperature phase transformations, offers a straightforward and effective method for producing various monophasic calcium phosphate phases. These include HA, brushite (dicalcium phosphate dihydrate), and monetite (dicalcium phosphate anhydrous) [25,26,27]. The 3D-printed calcium phosphate materials created through this method are notable for their high porosity, low crystallinity, biodegradability, and bioactive properties. These calcium phosphate-based materials have been clinically proven to be safe and effective as bone grafts for alveolar ridge preservation [28] and for personalized bone block grafts with precisely tailored shapes that fit the specific anatomical defects of individual patients [29]. The dual functionality of these 3D-printed bone grafts, which are capable of both drug release and bone regeneration, has also been demonstrated in laboratory and clinical investigations [30,31,32].

In this study, we aimed to overcome the limitations of previous OCP fabrication methods by introducing a two-step binder jetting approach that employs calcium sulfate-based powders in combination with a controlled low-temperature phase transformation. This strategy enables the direct fabrication of polymer-free, monophasic 3D-printed OCP constructs with significantly reduced processing time and enhanced phase purity, offering a more efficient and clinically relevant route for bone graft scaffold production. The objective of this study was to evaluate the feasibility of employing binder jetting technology with calcium sulfate-based powders, coupled with a controlled low-temperature phase transformation process, as a previously unreported and efficient method for fabricating monophasic OCP constructs. Specifically, we aimed to determine whether this process could achieve complete OCP phase transformation in a shorter timeframe than previous binder jetting techniques while maintaining desirable structural and biological properties. To this end, we conducted a characterization of the fabricated constructs, including assessments of phase composition, microstructure, surface wettability, mechanical strength, in vitro resorption behavior, and biological performance (cytotoxicity, cell proliferation, and osteogenic differentiation). The ultimate goal was to determine the suitability of this method for fabricating functional 3D-printed OCP constructs for bone grafting and related biomedical applications.

## 2. Results

Table 1 presents a summary of the phase composition percentages of the as-printed calcium sulfate samples after single-step transformation under various conditions. Using pH 7 and 37 °C, the phase transformation efficacy was low, and only brushite and non-transformed calcium sulfate (bassanite) were seen. Increasing the pH to 9 while maintaining a temperature of 37 °C could transform the calcium sulfate to OCP; however, the OCP content remained limited, equivalent to that of the non-transformed calcium sulfate (bassanite and gypsum). When the temperature was increased to 65 °C, nearly all calcium sulfate was transformed to either monetite or OCP. Using pH 9 was found to be more effective than pH 7 in obtaining OCP as the major phase, along with monetite and calcium sulfate as minor phases. When the temperature was further increased to 100 °C, calcium sulfate was transformed to monophasic HA at pH 9 and HA with monetite as a minor phase at pH 7. In summary, using pH 9, 65 °C yielded the greatest content of OCP in a single-step process.

**Figure 1:**
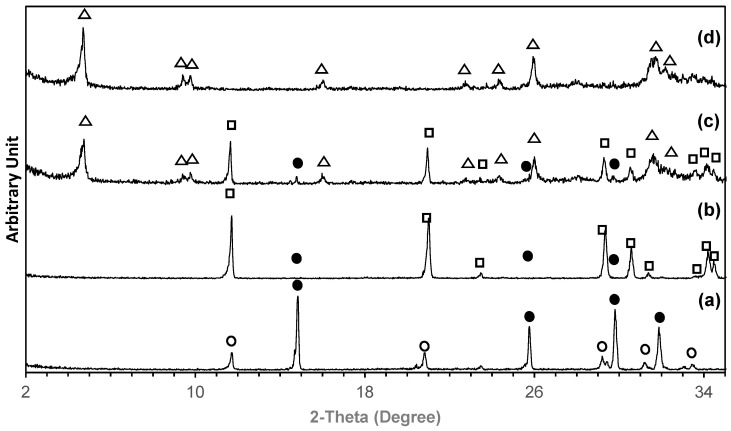
XRD pattern of 3D printed samples: (**a**) as-printed; (**b**) after being converted to brushite; after being converted to OCP using a pH of 8 and a temperature of 65 °C for 24 h (**c**) and 48 h (**d**). Symbols: •—bassanite; o—gypsum; **□**—brushite; **Δ**—OCP.

Table 2 summarizes the phase composition percentage of the as-printed calcium sulfate samples after the two-step transformation at 24 h and 48 h. At 24 h, the OCP content reached 90.4%, while 100% OCP was achieved after 48 h of transformation. Figure 1 presents the corresponding XRD patterns of samples after the two-step transformation process. Figure 1a shows the as-printed samples, which contain calcium sulfate phases, including bassanite (calcium sulfate hemihydrate) and gypsum (calcium sulfate dihydrate). After the first step of conversion at pH 6.5 and 37 °C for 48 h, the XRD pattern (Figure 1b) predominantly displayed peaks corresponding to brushite, with minor peaks indicating the presence of non-transformed bassanite. In the second step of conversion, conducted at pH 8 and 65 °C, the XRD pattern at 24 h (Figure 1c) revealed a mixture of OCP and brushite, with small residual peaks of bassanite. However, after 48 h (Figure 1d), only peaks corresponding to OCP were observed, indicating complete conversion.

**Figure 2:**
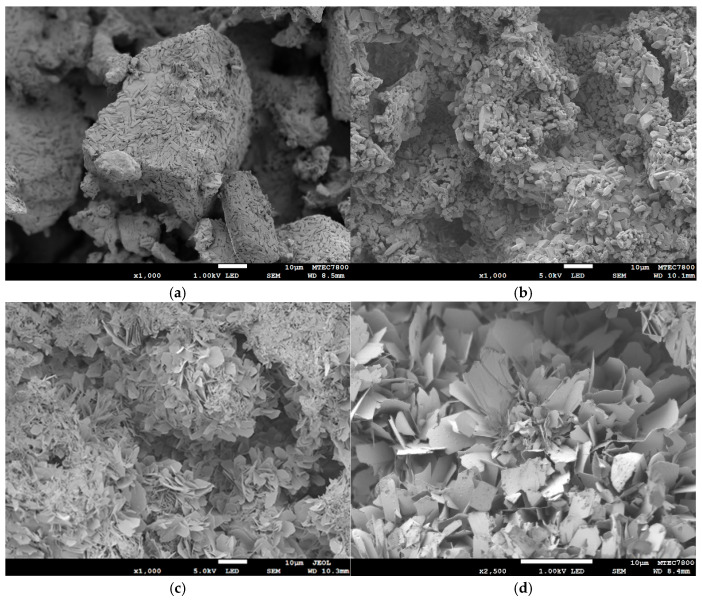
SEM images showing the microstructure of 3D printed sample before and after converting to brushite and then phase transformed to OCP using a pH of 8 and a temperature of 65 °C for 48 h: (**a**) as-printed, magnification ×1000; (**b**) after being converted to brushite, magnification ×1000; (**c**) after being converted to OCP, magnification ×1000; and (**d**) after being converted to OCP, magnification ×2500.

Figure 2 presents the representative microstructure of the sample before and after phase transformation to OCP using the two-step process. Figure 2a shows the as-printed calcium sulfate sample, which consists of large granules formed by intertwining primary rod-shaped crystals. These crystals are elongated and cylindrical, with well-defined edges and faces. Following the transformation to brushite (Figure 2b), the rod-shaped crystals of calcium sulfate are converted into thick, plate-like brushite crystals. After the subsequent transformation from brushite to OCP (Figure 2c,d), the microstructure predominantly exhibits a lamellar or thin-plate morphology characterized by layered, sheet-like structures that are typical of OCP crystals. Both the as-printed calcium sulfate sample and the converted samples exhibit a porous microstructure composed of clustered granules with a combination of macropores and micropores.

**Figure 3:**
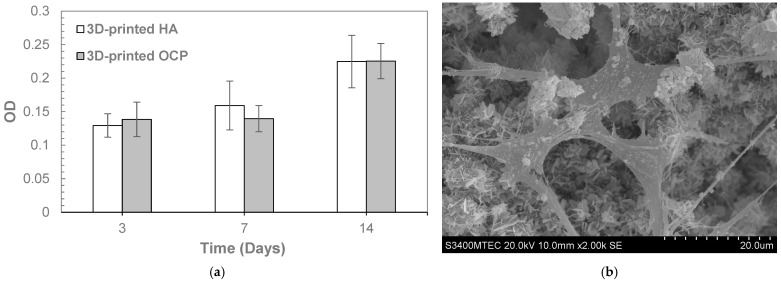
MC3T3-E1 cells proliferation by alamar blue assay: (**a**) OD (Mean ± SD, n = 6). No significant difference was observed between 3D-printed HA and 3D-printed OCP (*p* > 0.05).; (**b**) SEM image of cell morphology growth on the 3D-printed OCP sample at 14 days.

**Figure 4:**
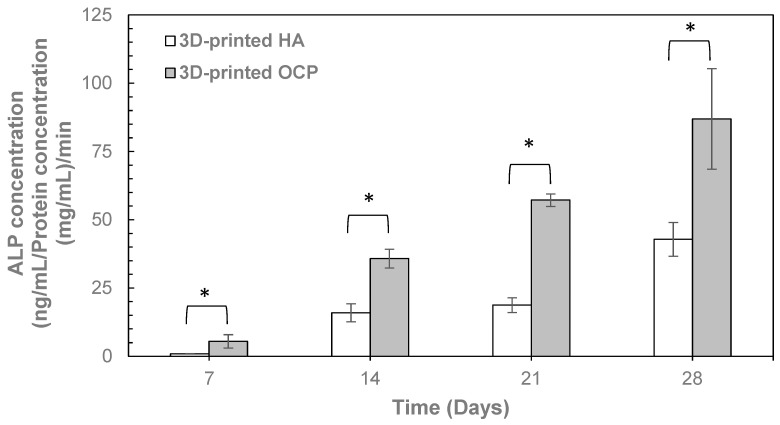
ALP activity of samples (Mean ± SD, n = 6). A significant difference (*) was observed between 3D-printed HA and 3D-printed OCP at all incubation times (*p* < 0.05).

**Figure 5:**
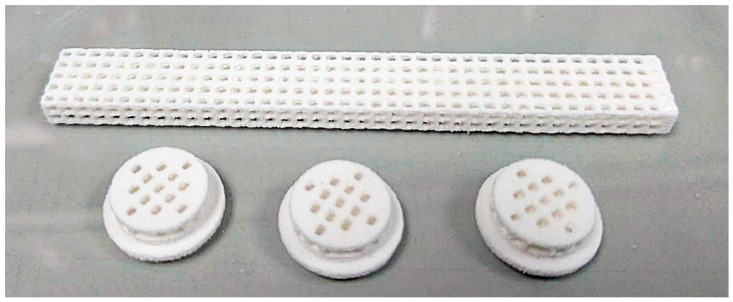
3D-printed OCP construct, demonstrating the shape and structure customization capability enabled by the approach developed in this study.

Given that monophasic OCP was successfully prepared through a two-step transformation process with a 48 h conversion time in the second step, the 3D-printed OCP samples fabricated under these conditions were selected for further characterization. After conversion, the compressive strength (Table 3) of the 3D-printed OCP was 7.65 ± 0.46 MPa, showing no significant difference compared to 3D-printed HA. The contact angle of 3D-printed OCP was measured as zero, similar to that of 3D-printed HA (Table 3), indicating the highly hydrophilic nature of both materials. In the evaluation of biocompatibility and bioactivity, the cell viability percentage, as determined by the MTT assay (Table 4), for the 3D-printed OCP was significantly lower than that of the negative control but significantly higher than for the 3D-printed HA. Nevertheless, all samples demonstrated cell viability above 70%, indicating no cytotoxic potential. Figure 3a shows the proliferation of MC3T3-E1 preosteoblast cells over various culturing periods by using the alamar blue assay. Cell growth on the surfaces of 3D-printed OCP and 3D-printed HA was comparable, with no significant difference observed. This was evidenced by a similar increase in optical density (OD) over time. Figure 3b depicts the sample surfaces after 14 days of MC3T3-E1 preosteoblast cell culture, revealing the typical cell morphology where the cells have grown, spread, and adhered to the sample surface. As shown in Figure 4, the ALP activity of both 3D-printed OCP and HA increased over time. Notably, 3D-printed OCP exhibited significantly higher ALP activity than HA at all time points. Figure 5 depicts a 3D-printed OCP fabricated using binder jetting and a two-step phase transformation of calcium sulfate, demonstrating the shape and structure customization approach developed in this study.

**Figure 6:**
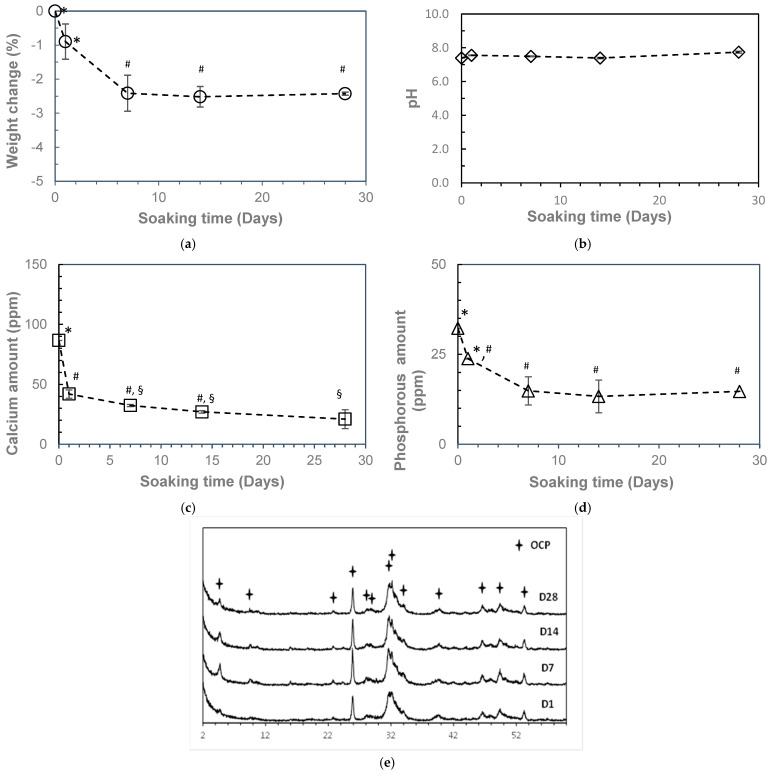
Resorbability of 3D printed OCP samples after soaking in pH 7.4 SBF at 37 °C for 1, 7, 14, and 28 days (Mean ± SD, n = 3): (**a**) weight loss; (**b**) pH; (**c**) calcium ions in SBF; (**d**) phosphorus ions in SBF; and (**e**) XRD patterns. Means that do not share a symbol (*, #, and §) are significantly different (*p* < 0.05).

**Figure 7:**
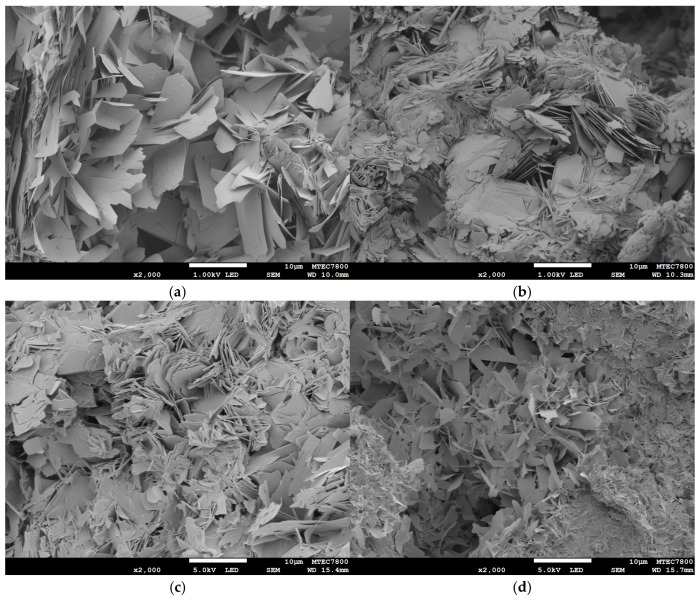
SEM images showing the microstructure of 3D-printed OCP samples after soaking in SBF at 37 °C for various periods: (**a**) 1 D; (**b**) 7 D; (**c**) 14 D; and (**d**) 28 D.

Figure 6 presents the resorption behavior of 3D-printed OCP during immersion in SBF (pH 7.4) at 37 °C over 28 days. The results indicate that the weight of the 3D-printed OCP decreased steadily until day 7, after which it stabilized, showing no further significant changes (Figure 6a). The pH of the SBF remained close to its initial value, with a slight increase observed over time, from 7.39 to 7.74, though this change was not statistically significant (Figure 6b). Furthermore, the concentrations of calcium and phosphate ions in the SBF declined progressively throughout the immersion period. A more rapid decrease was observed during the first 7 days, followed by a slower, nonsignificant decline thereafter (Figure 6c,d). XRD analysis reveals that the 3D-printed OCP sample immersed in SBF remained unchanged, retaining its original OCP phase throughout the immersion period. Figure 7 shows the SEM images of the sample before and after immersion in SBF. Although the crystals retained their lamellar or thin-plate morphology after immersion, their number and density increased after immersion in SBF, accompanied by some crystal growth. This indicates that there may be a growth and reprecipitation of OCP onto the original 3D-printed OCP sample.

## 3. Discussion

In this study, two experimental approaches were explored to fabricate monophasic OCP constructs through controlled phase transformation of 3D-printed calcium sulfate-based samples produced via binder jetting. The results demonstrated that a monophasic OCP structure could be successfully obtained using a two-step transformation process, whereas a direct single-step conversion from calcium sulfate was unsuccessful. When comparing calcium sulfate and brushite as precursors, the following simplified stoichiometric reactions describe the formation of OCP in disodium hydrogen phosphate solution:

8CaSO_4_·2H_2_O + 2HPO_4_^2−^ + 4PO_4_^3−^ → Ca_8_(HPO_4_)_2_(PO_4_)_4_·5H_2_O + 8SO_4_^2−^ + 11H_2_O

8CaHPO_4_·2H_2_O + 2HPO_4_^2−^ → Ca_8_(HPO_4_)_2_(PO_4_)_4_·5H_2_O + 4H_2_PO_4_^−^ + 11H_2_O

These reactions indicated the generation of H_2_PO_4_^−^ and SO_4_^2−^ into the medium, driving a measurable drop in pH for both pathways. Furthermore, the conversion of calcium sulfate to OCP demands both hydrogen phosphate (HPO_4_^2−^) and phosphate (PO_4_^3−^) ions in solution, whereas the transformation from brushite to OCP only requires HPO_4_^2−^. Consistent with these mechanisms, we observed a decrease in the pH of the disodium hydrogen phosphate solution after each phase-transformation step.

The higher solubility of brushite and structural compatibility with OCP would result in lower energy barriers for conversion. It transforms typically in the pH range of 6.0–7.0, often without requiring additional calcium or phosphate ions [33]. Calcium sulfate, however, is less soluble and largely pH-independent, significantly limiting the availability of calcium ions for OCP formation and necessitating an external phosphate source to enable conversion. Moreover, the released sulfate ions, which are not incorporated into OCP but remain free, can adsorb to calcium sites, creating an energy barrier for phosphate incorporation that can inhibit phosphate coordination, slowing OCP nucleation [34] and potentially leading to intermediate phases such as amorphous calcium phosphate (ACP) or brushite or monetite before OCP forms. Therefore, the reaction is thermodynamically favorable, but it is kinetically slow. Brushite and OCP share key structural features that greatly lower the barrier to direct phase transformation. By contrast, calcium sulfate must undergo complete sulfate-to-phosphate ion exchange and typically traverses several intermediates before OCP can nucleate. Consequently, calcium sulfate-based conversions are protracted and indirect, whereas brushite dissolves and reprecipitates as OCP under mild, near-physiological conditions, enabling a faster transformation.

Previous studies have demonstrated the direct conversion of calcium sulfate blocks (6 mm × 3 mm) into monophasic OCP [35,36]. However, conversion from brushite blocks has been shown to be superior, enabling the formation of phase-pure OCP with minimal incorporation of residual ions and significantly higher mechanical strength (diametral tensile strength (DTS) ≈ 6 MPa) compared to OCP derived from calcium sulfate (≈1 MPa) [35,37,38]. Moreover, because the transformation process is governed by a dissolution–precipitation mechanism that is both rate-limiting and dissolution-dependent, larger samples often develop pH and dissolution gradients across their thickness. These gradients impede complete transformation and promote the formation of secondary phases. In our study, the single-step conversion of 3D-printed calcium sulfate constructs (7 mm × 7 mm) resulted in incomplete transformation, yielding residual calcium sulfate, monetite, and brushite. In contrast, the two-step route, which first converts calcium sulfate to brushite and then transforms it to OCP, proceeded efficiently regardless of sample dimensions, achieving phase-pure OCP within approximately four days. The resulting constructs exhibited a compressive strength of 7.65 ± 0.46 MPa, which did not significantly differ from that of 3D-printed HA, a material successfully used as a bone graft in previous clinical trials, indicating sufficient mechanical strength for such applications [28,29,30]. This value is also comparable to previously reported compressive strengths of OCP fabricated using the binder jetting process [21,22]. However, the technique used in this study is substantially faster and more effective than earlier two-step methods using tricalcium phosphate precursors in binder jetting, which required at least 14 days and still yielded a mixture of OCP, brushite, and HA [21,22]. These findings underscore the distinct value of our approach, which combines the scalability of binder jetting with a tailored low-temperature transformation strategy to reliably produce high-purity, mechanically competent OCP constructs suitable for bone grafting. Unlike previously reported additive manufacturing techniques for OCP, which often rely on indirect methods such as incorporating OCP as a filler in polymer-based matrices [17,18,19] (e.g., DLP, DIW, or involves high-temperature sintering that destabilizes the OCP phase, our method enables the direct, polymer-free fabrication of bulk OCP structures under mild conditions. This represents a significant advancement in the scalable production of resorbable calcium phosphate materials for clinical bone grafting applications.

The contact angle of 3D-printed OCP was measured to be zero, comparable to that of 3D-printed HA, demonstrating the highly hydrophilic nature of both materials. Wettability and protein adsorption are crucial factors influencing cellular behavior during bone regeneration [39]. Hydrophilic surfaces promote the adsorption of adhesion-facilitating proteins such as fibronectin, which enhances osteoblast attachment, spreading, and differentiation, thereby supporting bone healing. In contrast, hydrophobic surfaces tend to adsorb proteins like albumin, which are less favorable for osteoblast adhesion. The type, conformation, and distribution of adsorbed proteins, governed by surface wettability and chemistry, form a bioactive interface that directs cellular responses. Moreover, improved wettability aids blood clot formation and angiogenesis, essential processes in early bone repair, while also stimulating osteoclast resorption and osteoblast proliferation, thus facilitating bone remodeling [40,41,42]. Although wettability and protein adsorption are traditionally considered surface phenomena, the high wettability observed in 3D-printed OCP and 3D-printed HA is also influenced by their intrinsic porous morphology, which enables rapid liquid absorption throughout the sample. Wettability describes not only how a liquid spreads on a surface but also how it penetrates a solid. In porous materials, the extensive internal pore surfaces significantly contribute to overall wettability due to their large surface area and complex structure. Consequently, porous materials often exhibit enhanced wettability compared to non-porous solids, as fluids infiltrate and wet internal pores, increasing the material’s affinity for liquids beyond the external surface. Similarly, protein adsorption and blood clotting occur not only on the external surface but also within the internal pore network [43], where the accessible surface area provides additional binding sites that improve cell attachment and biological responses critical for effective bone regeneration.

The resorbability of calcium phosphate-based biomaterials plays a critical role in their functionality as bone graft substitutes. SBF, which mimics the ionic concentration of human plasma, is widely used as an in vitro model to assess the bioactivity and degradation behavior of bone substitute materials. Studying the resorption of calcium phosphates in SBF provides preliminary insights into its chemical stability, phase transformation, and apatite-forming ability, which are considered early indicators of in vivo bioactivity [44]. The bioactivity of OCP was reported to arise from its relatively high solubility, which facilitates the release of calcium and phosphate ions, and its ability to undergo in situ transformation into HA, thereby supporting osteogenic processes [2,3,4,5]. Additionally, the higher solubility of OCP may contribute to bone regeneration by facilitating the creation of space for new tissue ingrowth. In this context, the resorption behavior of 3D-printed OCP was evaluated in SBF to provide preliminary evidence of its degradation profile and potential bioactivity.

In this study, 3D-printed OCP undergoes resorption in SBF, evidenced by weight loss as it dissolves and liberates Ca^2^⁺ and PO_4_^3−^ ions, which are vital for bone mineralization. This gradual dissolution underlies its bioresorbability, allowing the implant to be slowly replaced by native bone tissue. Although OCP is metastable in aqueous environments, it is generally expected to transform into the more stable HA phase due to the higher thermodynamic stability of HA compared to OCP [45]. OCP’s layered structure—comprising apatitic sheets that closely resemble HA, interspersed with hydrated layers—facilitates the epitaxial growth of HA crystals on the OCP surface [46,47,48]. This phase change proceeds via a dissolution–reprecipitation mechanism [26] and has been observed both under physiological conditions in vitro [49,50] and in vivo [51,52,53].

However, in our study, 3D-printed OCP remained unchanged after 28 days of immersion in SBF, showing no conversion to HA. This finding concurs with earlier reports [53,54,55], which likewise observed continued OCP growth rather than a phase change to HA. Although HA is thermodynamically more stable in SBF, OCP could preferentially nucleate and grow. Theoretical analyses of calcium-phosphate precipitation in SBF reveal that OCP’s nucleation rate far exceeds that of HA despite HA’s greater driving force for formation [45]. At 37 °C and pH 7, the free energy change for nucleation (ΔG) is −2.21 kJ mol^−1^ for OCP versus −6.24 kJ mol^−1^ for HA [56], indicating a stronger thermodynamic push for HA. However, OCP’s heterogeneous nucleation rate (J) is approximately ten orders of magnitude higher than HA’s under physiological conditions [40]. Additionally, HA’s larger critical nucleus radius and higher activation energy further impede its nucleation relative to OCP [57]. Under the static conditions used in the resorbability tests, these kinetic advantages enable OCP to precipitate and grow without transforming into HA [10]. It was also speculated that magnesium ions in the SBF could also inhibit the transformation of OCP to HA [53].

It has been demonstrated that the hydrolysis of OCP in aqueous environments involves a dynamic ion exchange process characterized by the uptake of calcium ions from the surrounding solution and the simultaneous release of phosphate ions into the medium [58]. This ion exchange plays a crucial role in the phase transformation of OCP to HA, a more thermodynamically stable phase under physiological conditions. In vitro studies have further confirmed that during this hydrolysis process, calcium ions diffuse into the OCP crystal lattice, promoting structural rearrangement, while phosphate ions are concurrently released from the material into the solution [59]. In the present study, we observed a gradual decrease in the concentrations of both calcium and phosphorus ions in the SBF over time. This trend suggests a net uptake of these ions from the solution, which may not align strictly with the OCP-to-HA transformation pathway. Instead, this behavior is indicative of a dissolution–reprecipitation mechanism, in which OCP continuously dissolves and reprecipitates, likely as fresh OCP rather than transforming completely into HA. Such a process implies that the material surface is in a dynamic equilibrium with the surrounding solution, allowing for localized reprecipitation of calcium phosphate phases. This cycling may be driven by changes in local ion concentrations, pH, and saturation levels, which are typical in static in vitro environments. The absence of detectable HA formation and the concurrent depletion of both calcium and phosphorus ions from the SBF may reflect a scenario in which the reprecipitated phase remains as OCP or as an intermediate calcium-deficient apatite rather than fully converting into crystalline HA. This hypothesis aligns with previous findings suggesting that the OCP to HA transformation is highly sensitive to environmental factors such as ion concentration, temperature, pH, and the presence of nucleating agents [49].

Although 3D-printed OCP did not transform to HA or form an apatite layer on its surface after immersion in SBF, this does not preclude 3D-printed OCP from its bioactivity for bone regeneration. It is important to note that the resorption behavior of OCP is also influenced by the type of immersion solution used, as different media can affect dissolution rates, ion exchange, and phase transformation kinetics [60]. In addition, the bioactivity of OCP arises from multiple mechanisms beyond just HA transformation or apatite formation. The complex in vivo environment, featuring dynamic fluid flow, immune cells, matrix proteins, and bone morphogenetic signals, can accelerate OCP degradation and conversion to apatite far beyond what is observed in static, cell-free SBF tests. It was reported that OCP actively interacts with cells by releasing calcium and phosphate ions through partial dissolution, which stimulates osteoblastic activity and bone regeneration. Studies have shown that OCP enhances ALP activity and promotes osteoblast proliferation by modulating key signaling pathways, such as the P38/MAPK, AKT, and JNK pathways, which are critical for cell survival, differentiation, and mineralization [61]. This cellular response occurs independently of an apatite layer. OCP is also known to be a precursor phase that can hydrolyze into calcium-deficient hydroxyapatite in vivo. Still, this transformation may be slow or incomplete in SBF under certain conditions, resulting in no phase transformation to HA or detectable apatite layer formation during in vitro testing [8,62]. Despite this, the biological effects of OCP remain potent due to its ionic dissolution and direct cell signaling effects. The physiological environment comprises enzymes, proteins, and cells that actively remodel and convert OCP, processes that are not fully replicated in SBF.

In this study, initial in vitro biological assessments, including MTT assays for cytotoxicity, alamar blue assays for cell proliferation, and ALP activity for osteogenic differentiation potential, were conducted to evaluate the biological performance of 3D-printed OCP. The results demonstrated that the 3D-printed OCP showed no cytotoxic potential and supported the proliferation of preosteoblast cells to a degree comparable with that of 3D-printed HA, suggesting its potential as an osteoconductive material capable of supporting cell attachment and growth on its surface. Moreover, the observed time-dependent increase in ALP activity for the 3D-printed OCP suggests its capacity to induce osteogenic differentiation, a critical factor for bone regeneration [61,63]. Notably, the ALP activity of 3D-printed OCP was higher than that of 3D-printed HA, further indicating superior osteogenic potential. These findings are consistent with previous studies that have highlighted the enhanced bioactivity of OCP in promoting osteoblastic differentiation [61,63].

A limitation of this study is that the in vitro resorption behavior and phase transformation of 3D-printed OCP have not been sufficiently characterized. SBF-induced apatite formation is a valuable, but not exclusive, indicator of bioactivity, particularly for materials like OCP, whose biological performance relies on cell-mediated processes. A comprehensive assessment of bioactivity requires a combination of SBF testing, in vitro biological assays, and in vivo evaluations. To better elucidate the osteogenic pathway of 3D-printed OCP, further investigations are warranted. These should include in vitro studies using alternative immersion solutions, as well as biological assays examining key osteogenic markers such as osterix (OSX), osteopontin (OPN), and osteocalcin (OCN). Additionally, in vivo animal studies and future clinical trials will be crucial for evaluating the material’s biocompatibility, long-term functionality, and capacity for integration with native bone tissue. Another limitation is the absence of quantitative surface roughness analysis, which plays a key role in influencing cellular behavior and protein adsorption. Although this parameter was not assessed in the current study, future studies should consider surface roughness characterization to complement the biological data and provide a more comprehensive understanding of the structure–function relationships of 3D-printed OCP.

## 4. Materials and Methods

### 4.1. Raw Materials and Fabrication

Calcium sulfate-based powder (VisiJet PXL Core, 3D Systems, Rock Hill, SC, USA) was loaded into a binder jetting 3D printer (Projet160, 3D Systems, Rock Hill, SC, USA) to fabricate cylindrical specimens measuring 7 mm in diameter and height for phase composition, compression testing, and microstructural analysis. Additionally, disc-shaped specimens with a diameter of 10 mm and a thickness of 2 mm were printed to evaluate contact angle, surface roughness, resorbability, biocompatibility, and bioactivity. The printing process utilized an aqueous-based binder (VisiJet PXL Clear, 3D Systems, Rock Hill, SC, USA) that adhered to the powder and solidified through evaporation, eliminating the need for curing. No post-processing was performed.

### 4.2. Phase Transformation

**Figure 8:**
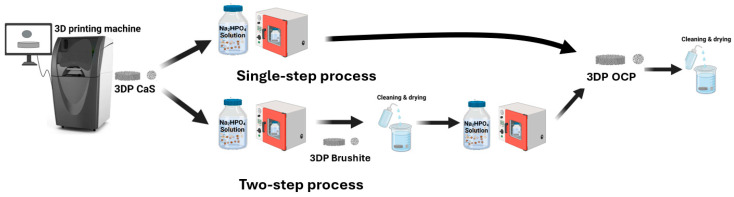
The fabrication process of 3D-printed OCP. Three-dimensional-printed OCP was prepared using binder jetting 3D printing combined with a phase transformation process, either in a single-step or two-step process.

The 3D-printed samples were experimentally transformed into OCP using either a single-step or two-step transformation process (Figure 8). In the case of the single-step process, the printed samples were directly converted to OCP by immersing in 1.0 M disodium hydrogen phosphate solution (Sigma Aldrich, St. Louis, MI, USA) for 120 h at various conditions, including pH (6.5 and 9.0) and temperature (37 °C, 65 °C and 100 °C). In the two-step process, the printed samples were initially converted to brushite by immersing them in a 1.5 M disodium hydrogen phosphate solution (Sigma Aldrich, St. Louis, MI, USA) at pH 6.5 and 37 °C for 48 h. After this treatment, the specimens were rinsed with distilled water and dried in an oven. Subsequently, the brushite-converted samples were further transformed into OCP by immersing them in a disodium hydrogen phosphate solution of the same concentration, adjusted to pH 8.0, and heating to 65 °C for up to 48 h. Following this second immersion, all samples were washed again with distilled water and then oven-dried. Three-dimensional-printed HA, which has been extensively studied in both laboratory and clinical investigations as a promising bone graft material [25,28,29,30,31,32], was prepared as a comparative sample by immersing 3D-printed calcium sulfate samples in 1.0 M disodium hydrogen phosphate solution (Sigma-Aldrich, St. Louis, MO, USA) at 100 °C for 48 h, washed with distilled water and oven-dried, following a previously reported protocol [25].

### 4.3. Characterizations

The phase composition of the samples was analyzed using an X-ray diffractometer (XRD, Rigaku TTRAX III, The Woodlands, TX, USA) equipped with a Cu Kα radiation source (wavelength = 0.15406 nm), operating at 300 mA and 50 kV. Measurements were carried out over a 2θ range of 2° to 35°, with a scanning speed of 3° per minute and a step size of 0.02°. The resulting diffraction patterns were processed and interpreted using JADE software version 9.7, which facilitated phase identification by comparison with entries from the International Centre for Diffraction Data (ICDD) database, including OCP: 00-026-105, bassanite: 01-080-795, monetite: 04-009-3755, brushite: 04-013-334, gypsum: 00-033-031 and HA: 01-089-4405. The phase content percentage was then calculated by using the Rietveld refinement method (JADE software version 9.7). Surface wettability was evaluated using the contact angle method with a goniometer (Model 200, Rame-Hart Instrument Co., Succasunna, NJ, USA), employing deionized water as the test liquid. Compression tests were performed on a universal testing machine (AGX-100kNV, Shimadzu, Kyoto, Japan) at a constant crosshead speed of 1.0 mm min^−1^. All the tests were carried out at 23 °C and 50% RH using five specimens. Compression strength was determined. Microstructures of the sample were carried out using a scanning electron microscope (SEM, JEOL JSM-7800F Prime, Akishima, Japan) at an accelerating voltage of 1 or 5 kV. Prior to SEM observation, the samples were dried at 80 °C for 24 h and subsequently sputter-coated with gold under vacuum to prevent charging and improve electrical conductivity.

### 4.4. Biocompatibility and Bioactivity Evaluation

#### 4.4.1. Cytotoxicity

A suspension of L929 fibroblast cells (NCTC clone 929 [L cell, L-929, derivative of Strain L], ATCC CCL-1, ATCC, Manassas, VA, USA) was prepared at a concentration of 1 × 10^5^ cells/mL in MEM (Gibco, Thermo Fisher Scientific, Waltham, MA, USA) and incubated at 37 °C for 24 h. The 3D-printed OCP samples were then extracted in 1 mL of MEM at 37 °C for 24 h. Following extraction, 100 µL of the extract was combined with 200 µL of the cell suspension in each well of a 96-well plate and incubated at 37 °C for another 24 h. Cell viability was assessed using the MTT assay (Invitrogen, Thermo Fisher Scientific, Waltham, MA, USA), with three replicates per sample. After incubation, the media was removed, and 100 µL of MTT solution (0.5 mg/mL) was added to each well. Plates were incubated in a CO_2_ incubator for 2 h in the dark. After incubation, the MTT solution was removed and replaced with 100 µL of DMSO (Sigma Aldrich, St. Louis, MI, USA) per well. Absorbance measurements were taken at 570 nm and 600 nm using a Biochrom Asys UVM340 microplate reader (Biochrom Ltd., Cambridge, UK), and optical density (OD) values were recorded. Control groups comprised a negative control (Nunc™ Thermanox™ Coverslips, Thermo Fisher Scientific, Waltham, MA, USA), a positive control (polyurethane film containing 0.1% Zinc diethyldithiocarbamate (ZDEC): RM-A, Hatano Research Institute, Kanagawa, Japan), and a blank control (wells without cells) to ensure the accuracy and reliability of the results. OD values, which measure light absorption by formazan crystals produced during mitochondrial reduction in live cells, indicate the degree to which a refractive medium slows transmitted rays of light. These measurements serve as a direct indicator of metabolic activity, with OD magnitude correlating linearly with the number of metabolically active cells present. The OD readings were subsequently used to calculate the percentage of cell viability.

% Cell viability = 100 × OD 570/OD 570_blank_

#### 4.4.2. In Vitro MC3T3-E1 Cell Proliferation

The samples were pre-soaked overnight in α-MEM completed medium (Gibco, Thermo Fisher Scientific, Waltham, MA, USA) and then placed into a 48-well plate. MC3T3-E1 preosteoblast cells (Subclone 4, ATCC CRL-2593, Manassas, VA, USA) were seeded onto the sample surfaces at a density of 5 × 10^5^ cells/mL by adding 100 µL per well, resulting in 5 × 10^4^ cells per sample. The cultures were maintained in a CO_2_ incubator at 37 °C with 5% CO_2_ and 95% relative humidity. Cell proliferation on the samples was assessed at 3, 7, and 14 days using the Alamar Blue assay (Invitrogen, Thermo Fisher Scientific, USA). Prior to the assay, the culture medium was removed, and the samples were washed three times with Dulbecco’s Phosphate-Buffered Saline (Gibco, Thermo Fisher Scientific, Waltham, MA, USA). Then, 20 µL of Alamar Blue solution was added to each sample containing 200 µL of fresh culture medium, followed by incubation in the CO_2_ incubator for 2 h. Absorbance was measured at 570 and 600 nm using a microplate reader (ASYS UVM340, Biochrom Ltd., Cambridge, UK) to obtain OD values. OD measurements quantify the enzymatic reduction in resazurin to fluorescent resorufin in viable cells, providing a direct correlation between cellular metabolic activity and viability.

For cell morphology analysis, cells were fixed with 25% EM-grade glutaraldehyde solution (Electron Microscopy Sciences, Hatfield, PA, USA) for 2 h at room temperature. Samples were then dehydrated through a graded ethanol series (RCI Labscan Co., Ltd., Bangkok, Thailand) and subjected to critical point drying using liquid CO_2_ (CPD300, Leica Microsystems, Buffalo Grove, IL, USA). Prior to SEM observation, the samples were coated with a gold layer by sputtering. SEM imaging was performed using a JEOL JSM-7800F Prime microscope (JEOL Ltd., Akishima, Japan) to examine cell morphology on the sample surfaces.

#### 4.4.3. ALP Activity Assay

The osteogenic differentiation potential of 3D-printed OCP was assessed by measuring ALP activity using the SensoLyte^®^ pNPP ALP assay kit (AnaSpec, Fremont, CA, USA). Bone marrow-derived mesenchymal stem cells (BM-MSCs; 2 × 10^4^ cells/well) were seeded onto 3D-printed OCP in 96-well plates containing phenol red-free complete medium. After overnight adhesion, the medium was replaced with osteogenic differentiation medium [completed DMEM medium (Gibco, Thermo Fisher Scientific, Waltham, MA, USA) supplemented with 100 nM dexamethasone, 10 mM β-glycerophosphate (Sigma-Aldrich, St. Louis, MI, USA), and 50 µg/mL ascorbic acid (Sigma-Aldrich, St. Louis, MI, USA). Cultures were maintained at 37 °C in a 5% CO_2_ humidified incubator, with medium refreshed every 72 h. ALP activity was quantified on days 3, 7, 14, 21, and 28. Samples were transferred to fresh wells, and cells were lysed using a buffer containing 0.1 M glycine (VWR Chemicals BDH^®^, Radnor, PA, USA), 1% Nonidet P-40 (USB Chemical, Cleveland, OH, USA), 1 mM MgCl_2_ (Sigma-Aldrich, St. Louis, MI, USA), and 1 mM ZnCl_2_ (EMSURE^®^; Merck KgaA, Darmstadt, Germany; pH 9.6) for 20 min at room temperature. Lysates underwent three freeze–thaw cycles (−80 °C for 30 min, thawed at 25 °C for 20 min) followed by centrifugation (12,000× *g*, 4 °C, 10 min) to pellet debris. ALP activity in the supernatants was analyzed using a microplate reader (BioTek, Inc., Houston, TX, USA) by measuring absorbance at 405 nm, which reflects the rate of enzymatic substrate conversion. Total cellular protein was quantified using the Bradford assay (Bio-Rad Laboratories, Hercules, CA, USA). ALP activity values were derived by subtracting blank absorbance from sample readings, referenced against an ALP standard curve (0–10 ng/mL), and normalized to total protein concentration.

### 4.5. In Vitro Resorbability

The resorbability of samples was determined by measuring the weight loss percentage in liquid. The experimental samples were initially dried in the oven at 80 °C for 1 h before their initial weight (W1) was measured using a precision 4-digit balance (AB 204-S, Mettler-Toledo International Inc., Greifensee, Switzerland). They were then immersed in pH 7.4 SBF at 37 °C for up to 28 days. At 1, 7, 14, and 28-day intervals, the samples were taken out, rinsed with deionized water, and dried at 80 °C for 24 h. The dried samples were then re-weighed and recorded as dried weight after immersion (W2). The weight loss percentage was calculated by dividing the weight difference by the initial weight using the following equation

Weight loss percentage = ((W2 − W1)/W1) × 100.

The change in phase composition and microstructure of the soaked samples was also evaluated by using XRD and SEM, respectively. The released content of calcium (Ca) and phosphorus (P) ions into the SBF at each soaking period was determined by using an inductively coupled plasma atomic emission spectrometry (ICP-OES, Horiba Activa, Irvine, CA, USA). The change in pH during the resorption process was also measured by using a pH meter (UB-5, Denver Instrument, Denver, CO, USA)

### 4.6. Statistical Analysis

Data are expressed as mean ± standard deviation (SD). Normality was confirmed using the Shapiro–Wilk test, and differences among sample groups and across time points were analyzed with the Student’s T-test for pairwise comparison or one-way analysis of variance (ANOVA) followed by Tukey’s Honestly Significant Difference post hoc tests for multiple comparisons. Statistical significance was defined as *p* < 0.05.

## 5. Conclusions

This study demonstrated the successful and efficient fabrication of monophasic 3D-printed OCP constructs from 3D-printed calcium sulfate precursors via a two-step phase transformation process. Compared to previous methods, particularly those involving tricalcium phosphate precursors, this approach significantly reduces processing time and enables the formation of phase-pure OCP, even in larger constructs, thereby overcoming limitations associated with pH and ion gradients. The resulting 3D-printed OCP constructs exhibited adequate mechanical strength, comparable to that of clinically studied 3D-printed HA, and demonstrated favorable in vitro bioactivity, including SBF resorption, non-cytotoxicity, cell proliferation, and osteogenic differentiation potential. Future research should focus on comprehensive in vitro and in vivo evaluations, particularly examining resorption, bone integration, and long-term performance in physiological environments. Additionally, optimizing the scaffold architecture and incorporating bioactive molecules or growth factors may further enhance the regenerative capabilities of 3D-printed OCP for clinical bone graft applications.

## Figures and Tables

**Table 1:** Summary of phase composition percentage of 3D-printed calcium sulfate sample after phase transformation at 120 h using various temperatures and pH.

Temperature (°C)	pH		Phase Composition (%)					
	Initial	After	Bassanite	Gypsum	Brushite	Monetite	OCP	HA
37	7.0	6.9	79.8 ± 1.6	-	20.2 ± 1.0	-	-	-
	9.0	8.8	17.2 ± 0.9	26.2 ± 1.8	-	-	56.6 ± 4.7	-
65	7.0	6.5	9.8 ± 0.4	1.3 ± 0.1	-	29.8 ± 1.1	59.1 ± 3.9	-
	9.0	8.0	9.1 ± 0.5	-	-	1.4 ± 0.3	89.5 ± 6.5	-
100	7.0	6.3	-	-	-	1.3 ± 0.1	-	98.7 ± 5.5
	9.0	7.0	-	-	-	-	-	100

**Table 2:** Summary of phase composition percentage of 3D-printed calcium sulfate samples, which were first converted to brushite and then phase transformed into OCP by using a pH of 8.0 and a temperature of 65 °C for various times, including 24 h and 48 h.

Times	pH		Phase Composition (%)		
	Initial	After	Bassanite	Brushite	OCP
24	8.0	6.5	0.8	8.9	90.4 ± 4.5
48	8.0	7.2	-	-	100

**Table 3:** Contact angle and compressive strength of samples (Mean ± SD, n = 5). For all properties, no significant difference was observed between 3D-printed HA and 3D-printed OCP (*p* > 0.05).

Samples	Contact Angle (Degree)	Compressive Strength (MPa)
3D-printed OCP	0 ± 0.00	7.65 ± 0.46
3D-printed HA	0 ± 0.00	7.32 ± 0.54

**Table 4:** Cell viability percentage of the samples by using MTT assay (Mean ± SD, n = 3). Blank and positive samples are excluded from the comparison. Means that do not share a symbol (*, #, and §) are significantly different (*p* < 0.05).

Samples	% Viability
Blank	100.0 ± 0.00
Positive	0.0 ± 0.00
Negative	100.1 ± 0.87 *
3D-printed OCP	96.7 ± 0.18 ^#^
3D-printed HA	82.4 ± 1.91 ^§^

## Data Availability

The original contributions presented in this study are included in the article; further inquiries can be directed to the corresponding author.

## Abbreviations

OCP	Octacalcium Phosphate
ACP	Amorphous Calcium Phosphate
HA	Hydroxyapatite
XRD	X-ray Diffraction
SEM	Scanning Electron Microscopy
DNA	Deoxyribonucleic acid
VEGFA	Vascular Endothelial Growth Factor A
SBF	Simulated Body Fluid
DLP	Digital Light Processing
DIW	Direct Ink Writing
DTS	Diametral Tensile Strength
MEM	Minimum Essential Medium
α-MEM	Minimum Essential Medium Eagle-Alpha Modification
DMEM	Dulbecco’s Modified Eagle Medium
MTT	3-(4,5-Dimethylthiazol-2-yl)-2,5-Diphenyltetrazolium Bromide
ALP	Alkaline Phosphatase
BM-MSCs	Bone Marrow-derived Mesenchymal Stem Cells
DMSO	Dimethyl Sulfoxide
PBS	Phosphate-Buffered Saline
OD	Optical Density
P38/MAPK	P38 Mitogen-activated Protein Kinases
AKT	Protein Kinase B or PKB
JNK	Jun N-terminal Kinases

## References

[1] Kovrlija I., Locs J., Loca D. (2021). Octacalcium phosphate: Innovative vehicle for the local biologically active substance delivery in bone regeneration. Acta Biomater..

[2] Kim J., Kim S., Song I. (2024). Octacalcium phosphate, a promising bone substitute material: A narrative review. J. Yeungnam Med. Sci..

[3] Nakano T., Yamashita Y. (2020). Octacalcium phosphate: A promising material for tissue engineering. Acta Biomater..

[4] Suzuki O., Shiwaku Y., Hamai R. (2020). Octacalcium phosphate bone substitute materials: Comparison between properties of bio-materials and other calcium phosphate materials. Dent. Mater. J..

[5] Suzuki O., Hamai R., Sakai S. (2023). The material design of octacalcium phosphate bone substitute: Increased dissolution and osteogenicity. Acta Biomater..

[6] Yokoi T., Goto T., Hara M., Sekino T., Seki T., Kamitakahara M., Ohtsuki C., Kitaoka S., Takahashi S., Kawashita M. (2021). Incorporation of tetracarboxylate ions into octacalcium phosphate for the development of next-generation biofriendly materials. Commun. Chem..

[7] Saito S., Hamai R., Shiwaku Y., Hasegawa T., Sakai S., Tsuchiya K., Sai Y., Iwama R., Amizuka N., Takahashi T. (2021). Involvement of distant octacalcium phosphate scaffolds in enhancing early differentiation of osteocytes during bone regeneration. Acta Biomater..

[8] Suzuki O., Imaizumi H., Kamakura S., Katagiri T. (2008). Bone regeneration by synthetic octacalcium phosphate and its role in biological mineralization. Curr. Med. Chem..

[9] Komlev V.S., Fadeeva I.V., Shvorneva L.I., Fomin A.S., Barinov S.M., Ferro D. (2010). Synthesis of octacalcium phosphate by precipitation from solution. Dokl. Chem..

[10] Iijima M., Kamemizu H., Wakamatsu N., Goto T., Doi Y., Moriwaki Y. (1991). Precipitation of octacalcium phosphate at 37 °C and at pH 7.4: In relation to enamel formation. J. Cryst. Growth.

[11] Tripathi G., Miyazaki T. (2021). Spontaneous fabrication of octacalcium phosphate: Synthesis conditions and basic characterizations. Bull. Mater. Sci..

[12] Kovrlija I., Menshikh K., Marsan O., Rey C., Combes C., Locs J., Loca D. (2023). Exploring the formation kinetics of octacalcium phosphate from alpha-tricalcium phosphate: Synthesis scale-up, determination of transient phases, their morphology and biocompatibility. Biomolecules.

[13] O’Sullivan R., Kelly D., Insley G., Suzuki O. (2020). Synthesis methodologies options for large-scale manufacturer of octacalcium phosphate. Octacalcium Phosphate Biomaterials: Understanding of Bioactive Properties and Application.

[14] Choudhary R., Indurkar A., Rubenis K., Grava A., Dubnika A., Hurle K., Locs J. (2024). Ultrafast and reproducible synthesis of tailor-made octacalcium phosphate. ACS Omega.

[15] Fedotov A.Y., Komlev V.S. (2022). Review of Octacalcium Phosphate Materials for Bone Tissue Engineering. Inorg. Mater. Appl. Res..

[16] Zafar M.J., Zhu D., Zhang Z. (2019). 3D Printing of Bioceramics for Bone Tissue Engineering. Materials.

[17] Preobrazhenskiy I.I., Tikhonov A.A., Evdokimov P.V., Shibaev A.V., Putlyaev V.I. (2021). DLP printing of hydrogel/calcium phosphate composites for the treatment of bone defects. J. Ceram. Sci. Technol..

[18] Xu Z., Lin B., Zhao C., Lu Y., Huang T., Chen Y., Li J., Wu R., Liu W., Lin J. (2022). Lanthanum doped octacalcium phosphate/polylactic acid scaffold fabricated by 3D printing for bone tissue engineering. J. Mater. Sci. Technol..

[19] Kim J., Kim W., Lee W., Mertamani R.N., Yun K., Kim S., Kim S.-J. (2023). 3D printed OCP bone scaffold with alginate enhancing osteogenic differentiation in MG-63 cells. MRS Commun..

[20] Ziaee M., Crane N.B. (2019). Binder jetting: A review of process, materials, and methods. Addit. Manuf..

[21] Komlev V.S., Popov V.K., Mironov A.V., Fedotov A.Y., Teterina A.Y., Smirnov I.V., Bozo I.Y., Rybko V.A., Deev R.V. (2015). 3D printing of octacalcium phosphate bone substitutes. Front. Bioeng. Biotechnol..

[22] Bozo I.Y., Deev R.V., Smirnov I.V., Fedotov A.Y., Popov V.K., Mironov A.V., Mironova O.A., Gerasimenko A.Y., Komlev V.S. (2020). 3D printed gene-activated octacalcium phosphate implants for large bone defects engineering. Int. J. Bioprint..

[23] Rahman Z., Charoo N.A., Kuttolamadom M., Asadi A., Khan M.A., Faintuch J., Faintuch S. (2020). Printing of personalized medication using binder jetting 3D printer. Precision Medicine for Investigators, Practitioners and Providers.

[24] Clares A.P., Gao Y., Stebbins R., van Duin A.C.T., Manogharan G. (2022). Increasing density and mechanical performance of binder jetting processing through bimodal particle size distribution. Mater. Sci. Addit. Manuf..

[25] Suwanprateeb J., Suvannapruk W., Wasoontararat K. (2010). Low temperature preparation of calcium phosphate structure via phosphorization of 3D-printed calcium sulfate hemihydrate based material. J. Mater. Sci. Mater. Med..

[26] Srion A., Thammarakcharoen F., Suwanprateeb J. (2022). Fabrication of Monetite by a controlled phase transformation of three dimensionally printed calcium sulfate construct. Chiang Mai J. Sci..

[27] Srion A., Palanuruksa P., Thammarakcharoen F., Suwanprateeb J. (2020). Low temperature fabrication of brushite by powder-based three dimensional printing coupled with phase transformation process. Chiang Mai J. Sci..

[28] Kijartorn P., Wongpairojpanich J., Thammarakcharoen F., Suwanprateeb J., Buranawat B. (2022). Clinical evaluation of 3D printed nano-porous hydroxyapatite bone graft for alveolar ridge preservation: A randomized controlled trial. J. Dent. Sci..

[29] Mekcha P., Wongpairojpanich J., Thammarakcharoen F., Suwanprateeb J., Buranawat B. (2023). Customized 3D printed nano-hydroxyapatite bone block grafts for implant sites: A case series. J. Prosthodont. Res..

[30] Jiamton C., Apivatgaroon A., Aunaramwat S., Chawalitrujiwong B., Chuaychoosakoon C., Suwannaphisit S., Jirawison C., Iamsumang C., Kongmalai P., Sukvanich P. (2023). Efficacy and safety of antibiotic impregnated microporous nanohydroxyapatite beads for chronic osteomyelitis treatment: A multicenter, open-label, prospective cohort study. Antibiotics.

[31] Thammarakcharoen F., Srion A., Chokevivat W., Hemstapat R., Morales N.P., Suwanprateeb J. (2022). Development of polycaprolactone infiltrated anti-tuberculosis drug-loaded 3D-printed hydroxyapatite for localized and sustained drug release in bone and joint tuberculosis treatment. Chiang Mai J. Sci..

[32] Suwanprateeb J., Thammarakcharoen F., Phanphiriya P., Chokevivat W., Suvannapruk W., Chernchujit B. (2014). Preparation and characterization of antibiotic impregnated microporous nano-hydroxyapatite for osteomyelitis treatment. Biomed. Eng. Appl. Basis Commun..

[33] Mandel S., Tas A.C. (2010). Brushite (CaHPO_4_·2H_2_O) to octacalcium phosphate (Ca_8_(HPO_4_)2(PO_4_)_4_·5H_2_O) transformation in DMEM solutions at 36.5 °C. Mater. Sci. Eng. C Mater. Biol. Appl..

[34] Cao X., Harris W.G., Josan M.S., Nair V.D. (2007). Inhibition of calcium phosphate precipitation under environmentally-relevant conditions. Sci. Total Environ..

[35] Sugiura Y., Munar M.L., Ishikawa K. (2018). Fabrication of octacalcium phosphate block through a dissolution-precipitation reaction using a calcium sulphate hemihydrate block as a precursor. J. Mater. Sci. Mater. Med..

[36] Sugiura Y., Munar M.L., Ishikawa K. (2018). Fabrication of octacalcium phosphate foam through phase conversion and its histological evaluation. Mater. Lett..

[37] Sugiura Y., Ishikawa K. (2019). Fabrication of pure octacalcium phosphate blocks from dicalcium hydrogen phosphate dihydrate blocks via a dissolution–precipitation reaction in a basic solution. Mater. Lett..

[38] Sugiura Y., Ishikawa K. (2018). Effect of Calcium and Phosphate on Compositional Conversion from Dicalcium Hydrogen Phosphate Dihydrate Blocks to Octacalcium Phosphate Blocks. Crystals.

[39] Wei J., Igarashi T., Okumori N., Igarashi T., Maetani T., Liu B., Yoshinari M. (2009). Influence of surface wettability on competitive protein adsorption and initial attachment of osteoblasts. Biomed. Mater..

[40] Xu L.C., Siedlecki C.A. (2007). Effects of surface wettability and contact time on protein adhesion to biomaterial surfaces. Biomaterials.

[41] Shiu H.T., Goss B., Lutton C., Crawford R., Xiao Y. (2014). Formation of blood clot on biomaterial implants influences bone healing. Tissue Eng. Part B Rev..

[42] He W., Ding F., Zhang L., Liu W. (2025). In situ osteogenic activation of mesenchymal stem cells by the blood clot biomimetic mechanical microenvironment. Nat. Commun..

[43] Tran P.A. (2021). Blood clots and tissue regeneration of 3D printed dual scale porous polymeric scaffolds. Mater. Lett..

[44] Kokubo T., Takadama H. (2006). How useful is SBF in predicting in vivo bone bioactivity?. Biomaterials.

[45] Lu X., Leng Y. (2005). Theoretical analysis of calcium phosphate precipitation in simulated body fluid. Biomaterials.

[46] Mathew M., Takagi S., Ammon H.L. (1993). Crystal structure of calcium adipate monohydrate. J. Crystallogr. Spectrosc. Res..

[47] Nelson D.G.A., McLean J.D. (1984). High-resolution electron microscopy of octacalcium phosphate and its hydrolysis products. Calcif. Tissue Int..

[48] Iijima M., Tohda H., Moriwaki Y. (1992). Growth and structure of lamellar mixed crystals of octacalcium phosphate and apatite in a model system of enamel formation. J. Cryst. Growth.

[49] Horváthová R., Müller L., Helebrant A., Greil P., Müller F.A. (2008). In vitro transformation of OCP into carbonated HA under physiological conditions. Mater. Sci. Eng. C.

[50] Rau V., Fosca M., Komlev V.S., Fadeeva I.V., Albertini V.R., Barinov S.M. (2010). In Situ Time-Resolved Studies of Octacalcium Phosphate and Dicalcium Phosphate Dihydrate in Simulated Body Fluid: Cooperative Interactions and Nanoapatite Crystal Growth. Cryst. Growth Des..

[51] Suzuki O., Nakamura M., Miyasaka Y., Kagayama M., Sakurai M. (1991). Bone formation on synthetic precursors of hydroxyapatite. Tohoku J. Exp. Med..

[52] Suzuki O., Kamakura S., Katagiri T., Nakamura M., Zhao B., Honda Y., Kamijo R. (2006). Bone formation enhanced by implanted octacalcium phosphate involving conversion into Ca-deficient hydroxyapatite. Biomaterials.

[53] Ban S., Jinde T., Hasegawa J. (1992). Phase transformation of octacalcium phosphate in vivo and in vitro. Dent. Mater. J..

[54] Yokoi T., Kim I.Y., Ohtsuki C. (2012). Mineralization of calcium phosphate on octacalcium phosphate in a solution mimicking in vivo conditions. Phosphorus Res. Bull..

[55] Ito N., Kamitakahara M., Yoshimura M., Ioku K. (2014). Importance of nucleation in transformation of octacalcium phosphate to hydroxyapatite. Mater. Sci. Eng. C.

[56] Nelson D.G.A., Salimi H., Nancollas G.H. (1986). Octacalcium phosphate and apatite overgrowths: A crystallographic and kinetic study. J. Colloid Interface Sci..

[57] Nelson D.G.A., Barry J.C. (1989). High-resolution electron microscopy of nonstoichiometric apatite crystals. Anat. Rec..

[58] Brown W.E., Mathew M., Tung M.S. (1981). Crystal chemistry of octacalcium phosphate. Prog. Cryst. Growth Charact..

[59] Suzuki O., Yagishita H., Yamazaki M., Aoba T. (1995). Adsorption of bovine serum albumin onto octacalcium phosphate and its hydrolyzates. Cells Mater..

[60] Petrakova N.V., Teterina A.Y., Mikheeva P.V., Akhmedova S.A., Kuvshinova E.A., Sviridova I.K., Sergeeva N.S., Smirnov I.V., Fedotov A.Y., Kargin Y.F. (2021). In vitro study of octacalcium phosphate behavior in different model solutions. ACS Omega.

[61] Anada T., Sato T., Kamoya T., Shiwaku Y., Tsuchiya K., Takano-Yamamoto T., Sasaki K., Suzuki O. (2016). Evaluation of bioactivity of octacalcium phosphate using osteoblastic cell aggregates on a spheroid culture device. Regen. Ther..

[62] Jung Y., Kim J., Kim S., Chung S.H., Wie J. (2022). Multiple proliferation signaling pathways are modulated by octacalcium phosphate in osteoblasts. Int. J. Med. Sci..

[63] Sugai Y., Hamai R., Shiwaku Y., Anada T., Tsuchiya K., Kimura T., Tadano M., Yamauchi K., Takahashi T., Egusa H. (2025). Effect of octacalcium phosphate on osteogenic differentiation of induced pluripotent stem cells in a 3D hybrid spheroid culture. Biomimetics.

